# Toxoplasma gondii infection damages the perineuronal nets in a murine model

**DOI:** 10.1590/0074-02760200007

**Published:** 2020-09-11

**Authors:** Ywlliane da Silva Rodrigues Meurer, Ramayana Morais de Medeiros Brito, Valeria Palheta da Silva, Joelma Maria de Araujo Andade, Sarah Sophia Guedes Linhares, Antonio Pereira, Valter Ferreira de Andrade-Neto, Andrea Lima de Sá, Claudio Bruno Silva de Oliveira

**Affiliations:** 1 Universidade Federal da Paraíba, Programa de Pós-Graduação em Neurociência Cognitiva e Comportamento, João Pessoa, PB, Brasil; 2 Universidade Federal do Rio Grande do Norte, Programa de Pós-Graduação em Psicobiologia, Natal, RN, Brasil; 3 Universidade Federal do Rio Grande do Norte, Departamento de Microbiologia e Parasitologia, Laboratório de Biologia da Málaria e Toxoplasmose - LABMAT, Natal, RN, Brasil; 4 Universidade Federal do Pará, Instituto de Ciências da Sáude, Laboratório de Neuroplasticidade, Belém, PA, Brasil

**Keywords:** Toxoplasma gondii, perineuronal nets, Wisteria floribunda

## Abstract

**BACKGROUND:**

Behavioral and neurochemical alterations associated with toxoplasmosis may be influenced by the persistence of tissue cysts and activation of an immune response in the brain of *Toxoplasma gondii*-infected hosts. The cerebral extracellular matrix is organised as perineuronal nets (PNNs) that are both released and ensheath by some neurons and glial cells. There is evidences to suggest that PNNs impairment is a pathophysiological mechanism associated with neuropsychiatric conditions. However, there is a lack of information regarding the impact of parasitic infections on the PNNs integrity and how this could affect the host’s behavior.

**OBJECTIVES:**

In this context, we aimed to analyse the impact of *T. gondii* infection on cyst burden, PNNs integrity, and possible effects in the locomotor activity of chronically infected mice.

**METHODS:**

We infected mice with *T. gondii* ME-49 strain. After thirty days, we assessed locomotor performance of animals using the open field test, followed by evaluation of cysts burden and PNNs integrity in four brain regions (primary and secondary motor cortices, prefrontal and somesthetic cortex) to assess the PNNs integrity using *Wisteria floribunda* agglutinin (WFA) labeling by immunohistochemical analyses.

**FINDINGS AND MAIN CONCLUSIONS:**

Our findings revealed a random distribution of cysts in the brain, the disruption of PNNs surrounding neurons in four areas of the cerebral cortex and hyperlocomotor behavior in *T. gondii*-infected mice. These results can contribute to elucidate the link toxoplasmosis with the establishment of neuroinflammatory response in neuropsychiatric disorders and to raise a discussion about the mechanisms related to changes in brain connectivity, with possible behavioral repercussions during chronic *T. gondii* infection.

Toxoplasmosis is a parasitic infection caused by *Toxoplasma gondii*, a parasite of the Apicomplexa ^1^ grouped into 16 haplogroups from six ancestral clades^2^ and four of these strains are accepted as the main clonal strains.^3^ This parasite is capable of establishing a chronic infection, with tropism for the cerebral tissue where it develops tissue cysts.^4^ Its entry into the central nervous system (CNS), like any other pathogen, depends on its ability to cross the blood-brain barrier. There are three main possibilities of CNS invasion by *T. gondii*: paracellular crossing, transcellular crossing or the so-called “Trojan horse” mechanism.^5^

After the invasion, evidence of manipulation by the parasite in host cells is not fully understood. In the brain, during acute infection tachyzoites are mainly observed in glial cells;^6^ while in chronic infection cysts are more commonly seen in neurons.^5^ This is probably because neurons, unlike astrocytes and other glial cells, are not efficient at eliminating the parasite and, thus, provides a safe niche where the parasite remains using the host cell during a persistent and chronic infection.^7^

*Toxoplasma gondii* also sparks interest among researchers because of its unusual ability to affect brain physiology,^8^ ^,^ ^9^ modulating the host behavior^10^ ^,^ ^11^ and perhaps triggering neuropsychiatric disorders.^12^ The presence of tissue cysts has a direct impact on the brain, interfering in neurotransmission,^8^ ^,^ ^10^ and stimulating immune system mechanisms.^11^ Some researchers reported evidence strengthening a plausible hypothesis that histopathological, immunological, and neuromodulatory changes would be related to behavior alterations of *T. gondii*-infected rodents.^5^ ^,^ ^13^ ^,^ ^14^ ^,^ ^15^

In addition, there are consistent data showing that the proliferation of tachyzoites (due to early acute infection or cyst rupture) in the brain might be involved in the establishment of neuropsychiatric symptoms, including the onset of the schizophrenia.^15^ Although there is not a definitive causal ratio between *T. gondii* infection and schizophrenia, the imbalance of normal brain neurotransmitters such as dopamine, serotonin and glutamate may contribute to the disorder.^16^ ^,^ ^17^ Moreover, the neuroinflammatory response during chronic infection can also contribute to behavioral abnormalities or neuropsychiatric-like symptoms noticed in rodents and humans,^18^ ^,^ ^19^ as well as other behavioral alterations such as anxiety-like and depression.^20^ In addition, Bay-Richter et al.^21^ showed that increased levels of cytokines may be causally related to behavioral changes in rodents. Probably, the inflammatory mediators might be evoked by activation of brain cells (microglia, astrocytes), due to latent toxoplasmosis^18^ ^,^ ^22^ and these factors, including the release of matrix metalloproteinases (MMPs) in the brain^23^ could compromise the perineuronal nets (PNNs) structures in the brain.

PNNs are specialised and complex lattice-like extracellular matrix assemblies of chondroitin sulfate proteoglycans (CSPGs), tenascin-R, hyaluronan, and link proteins.^24^ These structures involve the cell body, dendrites, and proximal portion of the axon of distinct neuronal subpopulations,^25^ creating a supportive microenvironment for neurons and acting in the maintenance of neuronal plasticity and synaptic properties.^26^ There is evidence to suggest that disruption of PNNs is a pathological mechanism associated with neuropsychiatric disorders, such as Alzheimer’s disease, multiple sclerosis, bipolar disorder, and schizophrenia.^27^ ^,^ ^28^ Many factors can influence the PNNs integrity, such as the enzymatic activity of MMPs as a result of brain trauma and/or inflammation. It is possible to suggest that the susceptibility of PNNs to degeneration due to inflammatory stimuli can contribute to synaptic impairment and interfere in the CNS,^26^ ^,^ ^29^ which could contribute to behavioral abnormalities. In this context, we aimed to analyse the impact of *T. gondii* infection on cyst burden, PNNs integrity, and possible effects in the locomotor activity of chronically infected mice.

## MATERIALS AND METHODS

*Animals and Toxoplasma gondii infection* - Outbred Swiss Webster mice (6-8 weeks old and 20-25 g weight) were used in this study. Twelve male mice were randomly assigned to control (n = 6) and *T. gondii*-infected (n = 6) groups. *T. gondii* was orally infected following the infection protocol from Oliveira et al.^30^ Briefly, mice were infected with 25 *T. gondii* ME49 cysts which were maintained in Swiss Webster mice and isolated before infection. Both experimental groups were monitored for 30 days to establish chronic toxoplasmosis.^31^ The animals housed under controlled conditions of light, temperature and feeding (22 ± 2ºC, 12-h day/night cycle with food and water ad libitum). For immunohistochemical experiments, animals were euthanised and brains were collected for further processing. All experimental conditions were approved by The Federal University of Rio Grande do Norte Committee on the Use and Care of Animals (protocol number 043/2016).

*Open field test* - The locomotor activity of non-infected and *T. gondii*-infected mice was assessed using the open field test. We performed the test in a circular arena (37 cm of diameter and solid walls of 22.5 cm) with its an open top and floor divided into centre and peripheral zones. We gently placed each mouse in the centre of the open field arena and videotaped for 10 minutes. We scored the spontaneous locomotor activity while the animals freely moved around in the apparatus. We measured traveled distance and average speed using automated tracking by ANY-MAZE software (Stoelting, USA). The behavioral data collection was blind to the experimenter and the animal groups.

*Perfusion, histological procedures, and microscopy* - Thirty days after initial infection, mice were lethally anesthetised using a ketamine-xylazine cocktail (Ketamine 10%, 100 mg/mL and xylazine 2%, 20 mg/mL) and subsequently perfused transcardially with heparinized 0.1 M phosphate buffer (PBS; pH 7.2-7.4) for 10 min followed by ice-cold 4% paraformaldehyde (PFA) in 0.1 M phosphate buffer (PBS; pH 7.2-7.4) for 20 min. Brains were collected and embedded with OCT. Brains were removed from the skull, cryoprotected in 30% sucrose solution for 48 h. For immunolabeling, sections were cut at 100 µm thickness using a vibratome. Coronal brain sections through the prefrontal cortex (mPFC), primary motor area (M1), secondary motor area (M2) and somatosensory area (S1) were analysed according to a standard mice brain atlas.^32^ ^,^ ^33^ These areas were chosen for the analysis because they presented a high expression of PNNs.

The sections were stained to investigate PNNs using biotinylated *Wisteria floribunda* agglutinin (WFA) (1: 5000; Vector Laboratories, CA, USA) overnight at room temperature after that detected with avidin-biotin system incubation for 2 h and visualised by 3’,3’ diaminobenzidine precipitation. Sections were then mounted on glasses slides, followed by dehydration in alcohol solution series, cleared in xylenes, counterstained by Nissl and coverslipped with entellan (Merck, Darmstadt, Germany).

**Fig. 1:**
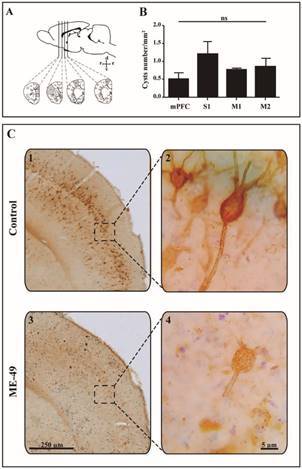
perineuronal nets (PNN+) *Wisteria floribunda* agglutinin (WFA)-labelled cells are strong reduced in different areas in brain of *Toxoplasma gondii*-infected mice. (A) Coronal slices were collected in the prefrontal area, somatosensory area (S1), primary and secondary cortices (M1 and M2, respectively); (B) *T. gondii* cysts quantification of the brain regions analysed. (C) Representative photomicrographs of PNN+ WFA-labelled cells in brain of control (C1, C2) and ME-49 (C3, C4) groups. Scale Bar = 250 µm (2.3x; low magnification) and 5 µm (100x; high manification). Data represent the mean ± standard error of the mean (SEM).

In view of we observed a strong reduction on WFA-labeling of brain structures of infected animals, we counted the WFA-labelled cells to avoid overestimated PNN damage from results of densitometric analysis. We perfomed the counting by means an unbiased stereological technique using Stereo Investigator software (v.10.51, MicroBrightField, Williston, VT). Briefly, contours in every section along rostrocaudal axis (divided in three levels) in the region of interest areas (mPFC, S1, M1 and M2) were determined at ×4.3 magnification, followed by randomly determination of counting frame in drawn contours using a Optiphot-2 microscope (Nikon Eclipse 80i, Japan) equipped with a motorised stage (MAC200, Ludl Electronic Products, Hawthorne, NY). Every three rostro caudal levels (rostral, median and caudal) throughout the region of interest was analysed, yielding a mean of six sections per brain area using a 100x oil immersion planapochomatic objective lens (NIKON, NA1.4, Nikon E600, Nikon, Tokyo, Japan) in order to detect and count unambiguously specimens in dissector probe low power. The cysts were counted in each coronal brain sections (e.g., mPFC, M1, M2 and S1) along WFA immunoreactivity analysis through bright field microscopy (Fig. 1A). The density score (cells/mm2) for WFA-positive cells per region interest was automatically generated by the optical fractionator formula of the Stereo Investigator software (MicroBrightField Japan, Tokyo, Japan).

*Data analysis and statistical procedures* - Statistical analysis and graphical images were carried out with SPSS (SPSS Inc., Chicago, IL, USA) and GraphPad Prism 6 software (GraphPad Software, La Jolla, CA, USA), respectively. The results are expressed by means ± standard error of the mean (SEM). Shapiro Wilk’s and Levene’s tests were applied to evaluate homogeneity and homoscedasticity of data records. One-way analysis of variance (one-way ANOVA) and Unpaired T-test were used when appropriate and as indicated in legends. Differences between infected and control groups were considered significant when ρ was less than 0.05.

## RESULTS

*Cysts counts* - In Fig. 1B, our data reveal higher cyst counts in somatosensory cortex (m = 1.52 ± 0.36), followed by M2 (m = 0.83 ± 0.23), next M1 (m = 0.75 ± 0.05), and mPFC (m = 0.45 ± 0.18). One-way ANOVA for the number of WFA-positive cells in brain revealed that somatosensory cortex exhibit more, but not significantly (t = 2.413; p = 0.103), cysts counts when compared to M1, M2 and mPFC areas.

**Fig. 2:**
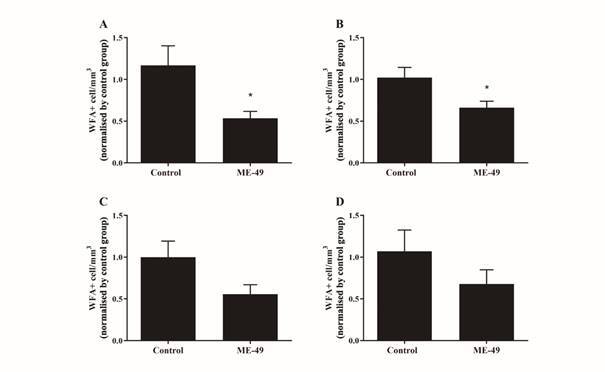
effects of *Toxoplasma gondii* infection on number of *Wisteria floribunda* agglutinin (WFA)+-labelled cells in the medial prefrontal cortex (mPFC; 2A), primary somatosensory cortex (S1; 2B), primary motor cortex (M1; 2C) and secondary motor cortex (M2; 2D). Data are expressed as the mean ± standard error of the mean (SEM). *: p < 0.05 vs. control group.

*PNN+ WFA-labelled cells* - We examined the PNN-WFA+-labelled cells in the brain of control or infected mice. As shown in Fig. 1A, the regions examined were the S1, mPFC, M1 and M2. In Fig. 1C, we observed a strong reduction on PNN-WFA+-labelled cells in brain structures of infected animals compared to control animals, which demonstrate a broad damage on extracellular matrix in neurons. Unpaired T-test reveals that the infection affects the number of WFA+-labelled cells in brain areas of infected animals (Fig. 2). In both mPFC (p = 0.036) and S1 (p = 0.026) areas were observed the lower number of PNN-WFA+-labelled cells in infected mice compared to the control group. In contrast, we do not observe differences in the M1 (p = 0.079) and M2 (p = 0.229) brain areas of infected animals compared to the control group. In addition, Unpaired Test-T reveals similar effects regarding PNN-WFA+ levels analysed by ROD in the brain areas of infected and control groups (data unshown). *T. gondii*-infected animals exhibit reduced PNN-WFA+ immunoreactivity in both mPFC (p = 0.022) and S1 (p = 0.004) than M1 (p = 0.150) or M2 (p = 0.134) brain areas compared to control group.

**Fig. 3:**
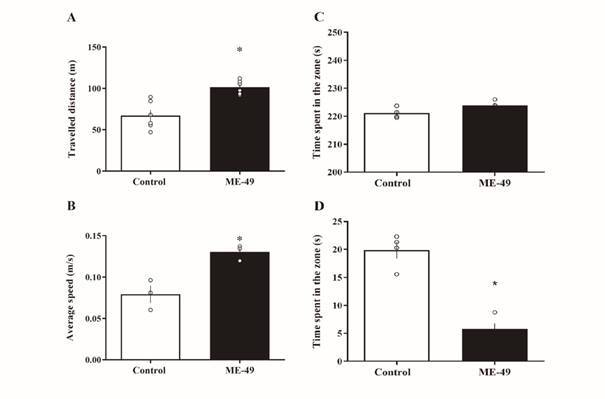
the open-field apparatus was used to assess locomotor activity. We analysed the distance travelled (A), average speed developed (B), and the time spent in the external (C) and in the centre zone (D) of the open field were recorded.by control and ME-49 infected mice during 10 minutes. Results were expressed as the mean ± standard error of the mean (SEM) of total distance travelled in meters and average speed in meters per second. *: p < 0.05 vs. control group.

*Locomotor activity* - As shown in Fig. 3A, the Unpaired T-test reveals there is a significant increase in the total distance traveled of the infected mice (p < 0.05) compared to control mice during the open field test. In addition, infected mice developed higher speed average in the arena (p < 0.05) than control mice (Fig. 3B). Finally, there was no differences in spent time in the external zone between *T. gondii*-infected and control mice (p = 0.075) of the open field (Fig. 3C). On the other hand, *T. gondii*-infected mice spent less time turn up in the centre zone (p = 0.002) compared to control (Fig. 3D).

## DISCUSSION

Searching for an explanation on how *T. gondii* could be acting on the disturbance of its host behavior, we analysed the distribution of tissue cysts and the integrity of PNNs in different brain areas: primary motor cortex, secondary motor cortex, primary somatosensory cortex, and medial prefrontal cortex. In this study, we demonstrated that there is no difference in *T. gondii* cysts distribution, despite the strong PNNs disruption observed in some brain areas. Also, we observed that chronic *T. gondii*-infection induces locomotor hyperactivity. *T. gondii* infected animals show greater traveled distance and average speed in the open field test compared to controls. Further, they also spent less time in the centre zone of the OF compared to uninfected animals.

As mentioned, our results do not show differences in cysts distribution in the brain of infected mice. Regarding this, our findings agree with others that reported the lack of tropism for specific brain regions.^34^ However, the role of tissue cysts in stimulating a chronic inflammatory environment should be highlighted, since the cyst burden could be indirectly affecting the brain homeostasis and functioning, as well as is directly correlated to neuronal impairment in specific brain areas and to high levels of inflammatory mediators.^35^ Importantly, the long life of brain cysts in the hosts has been suggested to produce a strong change in brain function through neurochemical modulation, which according to some authors may be related to changes in dopaminergic metabolism.^11^ ^,^ ^36^ The role of *T. gondii* in modulating dopamine production through its tyrosine hydroxylase-like protein^36^ can reinforce the possible link between neurotoxoplasmosis and the establishment of neuropsychiatric disorders, such as bipolar disorder, schizophrenia, and depression-like behavior. However, the relationship between *T. gondii* infection and behavioral changes may not be addressed exclusively by the dopaminergic neurotransmission manipulation,^37^ but also due to interference in the neuronal circuitry and structure.^38^

Many mechanisms, such as dysregulation of neurotransmitters signaling,^39^ changes in proteins of synaptic connectivity^40^ and neuroinflammation,^41^ ^,^ ^42^ induced by *T. gondii* could be the answer behind the behavioral changes observed in the infected hosts. Regarding the inflammatory profile during chronic infection, it is known that high levels of inflammatory cytokines are detrimental for cerebral integrity.^41^ Thus, it is possible that the constant inflammatory stimuli offered by the parasite could act compromising the brain cytoarchitecture like we have been suggesting,^43^ through impairing structural components that are crucial to maintaining the brain homeostasis.

PNNs are important to normal GABAergic connectivity.^44^ Previous studies suggest key roles to PNNs in neuronal plasticity and brain connectivity through to contribute to neuronal signaling, the regulation of synaptic formation, and stabilisation and is related to the proper expression of animal behavior.^26^ ^,^ ^27^ ^,^ ^45^ To verify the ability of *T. gondii* to contribute to the disruption of structural components of the brain, we analysed the number of PNNs surrounding neurons (PNN+ cells) in the four different cortex regions mentioned above. Here, we have shown that *T. gondii*-infected mice exhibited a marked reduction of PNN+ WFA-labeled cells in all four analysed brain areas. We showed the disruption of the most studied extracellular matrix component, the CSPG, labeled with *Wisteria floribunda* agglutinin in the prefrontal cortex, somesthetic, and motor cortices. These findings are particularly important related to neurotoxoplasmosis and neuropsychiatric disorders relationship, as it can be useful in the understanding of how the chronic infection could be interfering directly or indirectly in the brain functioning.

PNNs disruption was proposed to produce behavioral impairments through an imbalance in neural excitatory-inhibitory activity^46^ and may increase neuron vulnerability to oxidative stress^47^ and death.^48^ In fact, the PNNs disruption itself can be induced by neuroinflammatory events, increased reactive oxygen and nitrogen species production, and MMPs activation.^49^ Recently, previous studies reported increased neural apoptosis and decreased synapses in the brain of infected mice induced by excessive neuroinflammation and neurotransmitters dysfunctions.^42^ ^,^ ^50^ Interestingly, a recent study reported an imbalance between immune activation factor (IL1b, VCAM-1, IFNγ) and proteins related to tissue repair (TIMP-1, PAI-1, MMP2, MMP9) in the frontal cortex in the brain of chronically infected mice.^51^ Earlier studies proved that chronic *T. gondii* infection activates the NF-kB pathway and, in turn, induce excessive cytokine expression (e.g., TNF and IL-1β) and upregulation of MMP-9 in activated astrocytes and microglia, suggesting the ability of *T. gondii* to modulate the brain parenchyma and cerebral homeostasis.^50^ ^,^ ^52^

Several behavioral abnormalities during chronic *T. gondii* infection have been reported,^16^ ^,^ ^53^ ^,^ ^54^ such as avoidance of feline odor,^13^ ^,^ ^55^ ^,^ ^56^ increased activity,^57^ trappability^53^ ^,^ ^55^ and changes in exploratory and risk/unconditioned fear behavior.^20^ ^,^ ^37^ In this study, we demonstrated that chronically infected mice exhibited locomotor hyperactivity during the open field test. The increased locomotor activity and average speed observed here can be a result of the PNNs impairment, as an indirect effect of *T. gondii* infection. This hypothesis can be supported by data showing that experimental depletion of PNNs leads to an hyperlocomotor activity in mice^58^ and, thus, the disruption of PNNs may influence the function and morphology of neurons, leading to changes in the host’s brain activity and connectivity during chronic infection. Therefore, it is possible that brain damage and hyperlocomotion induced by *T. gondii* would be associated with persistent stimulation of inflammatory mediators and neurotransmitters dysregulation, which could contribute to PNNs impairment, during chronic infection.

*In conclusion* - In summary, our results taken together reinforce the behavioral changes and absence of neurotropism along of the *T. gondii*-chronic infection. Moreover, despite further investigations that are needed, we hypothesised that perineuronal disruption induced by *T. gondii* would be associated with persistent modulation of the neuroimmunological system and unbalance of tissue repair mechanisms. These data can be useful to the understanding of possible mechanisms behind behavioral changes observed in the infected host. In this context, the disrupted PNNs emerge as an additional neuropathological mechanism that contributes to compromise the cerebral integrity and excitatory-inhibitory circuitries during chronic infection in the hosts.
